# The Efficacy of a Web-Based Stress Management Intervention for Employees Experiencing Adverse Working Conditions and Occupational Self-efficacy as a Mediator: Randomized Controlled Trial

**DOI:** 10.2196/40488

**Published:** 2022-10-20

**Authors:** Patricia Nixon, David Daniel Ebert, Leif Boß, Peter Angerer, Nico Dragano, Dirk Lehr

**Affiliations:** 1 Department of Health Psychology and Applied Biological Psychology Institute of Psychology Leuphana University Lueneburg Germany; 2 Division of Psychology & Digital Mental Health Care Department for Sport & Health Sciences Technical University of Munich Munich Germany; 3 Faculty of Medicine Institute of Occupational, Social and Environmental Medicine Centre for Health and Society, Heinrich Heine University Duesseldorf Germany; 4 Institute of Medical Sociology Centre for Health and Society University Hospital Duesseldorf Germany

**Keywords:** occupational eMental health, stress, occupational self-efficacy, effort-reward imbalance, randomized controlled trial

## Abstract

**Background:**

Work stress is highly prevalent and puts employees at risk for adverse health consequences. Web-based stress management interventions (SMIs) promoting occupational self-efficacy might be a feasible approach to aid employees to alleviate this burden and to enable them to improve an unbalanced situation between efforts and rewards at work.

**Objective:**

The first aim of this randomized controlled trial was to investigate the efficacy of a web-based SMI for employees perceiving elevated stress levels and an effort-reward imbalance in comparison to a waitlist control (WLC) group. Second, we investigated whether the efficacy of an SMI could be explained by an increase in occupational self-efficacy and whether this personal resource enables employees to change adverse working conditions.

**Methods:**

A total of 262 employees reporting effort-reward imbalance scores over 0.715 and elevated stress levels (10-item Perceived Stress Scale [PSS-10] score ≥22) were randomly assigned to either the intervention group (IG; SMI) or the WLC group. The primary outcome was perceived stress measured using the PSS-10. The secondary outcomes included mental and work-related health measures. Four different mediation analyses were conducted with occupational self-efficacy, efforts, and rewards as mediators. After eligibility screening, data were collected web based at baseline (T1), 7 weeks (T2) and 6 months (T3).

**Results:**

Study participation was completed by 80% (105/130, 80.8%) in the IG and 90% (119/132, 90.2%) in the WLC group. Analyses of covariance revealed that stress reduction was significantly higher for the SMI group compared with the WLC group at T2 (d=0.87, 95% CI 0.61-1.12, P<.001) and T3 (*d*=0.65, 95% CI 0.41-0.90, P<.001). Mediation analyses indicated that occupational self-efficacy mediated the beneficial effect of the SMI on stress directly. Furthermore, the analyses revealed a significant indirect effect of occupational self-efficacy via rewards (*b*=0.18, t_259_=4.52, P<.001), but not via efforts (b=0.01, t_259_=0.27, P>.05) while efforts still had a negative impact on stress (*b*=0.46, t_257_=2.32, P<.05).

**Conclusions:**

The SMI was effective in reducing stress and improving occupational self-efficacy in employees despite them experiencing an effort-reward imbalance at work. Results from mediation analyses suggest that fostering personal resources such as occupational self-efficacy contributes to the efficacy of the SMI and enables employees to achieve positive changes regarding the rewarding aspects of the workplace. However, the SMI seemed to neither directly nor indirectly impact efforts, suggesting that person-focused interventions might not be sufficient and need to be complemented by organizational-focused interventions to comprehensively improve mental health in employees facing adverse working conditions.

**Trial Registration:**

German Clinical Trials Register DRKS00005990; https://tinyurl.com/23fmzfu3

## Introduction

More than a decade ago, the World Health Organization identified stress as major risk factor for adverse consequences on physical and mental health for the 21st century [1]. In particular, the workplace can be a source of stress that can be associated with an increased risk of depression and cardiovascular diseases [2-4]. Next to such harmful effects on employees’ personal lives and health, experiencing high strain at work can entail substantial societal costs [5].

One of the most prominent theoretical frameworks to investigate workplace stressors is the effort-reward imbalance model [6]. In short, this model is based on the premise of an imbalance between efforts invested and low rewards received in return. Both efforts and rewards therefore reflect subjectively perceived working conditions employees are exposed to. Rewards can be distinguished between financial payments, job security, or career prospects, and intangible compensation such as esteem or praise. During the past few decades, the model was well researched. It was shown that employees experiencing an effort-reward imbalance have an increased risk of depression [7], lower immunity [8], or coronary heart disease [9]. Multiple systematic reviews demonstrated robust evidence for the links between an effort-reward imbalance and health, and suggested that it can instigate psychological, physical, and behavioral health–impairing pathways [10-12].

Psychosocial hazards were identified as one of the key emerging health risks [13] and there were significant developments to address psychosocial risk factors at work. For example, the National Standard of Canada for Psychological Health and Safety in the Workplaces provides a comprehensive framework for an approach to ensure a psychologically healthy workplace [14]. Notably, changing adverse working conditions requires a timely and complex transformational process that can be a considerable source of work stress itself and is associated with different risks such as an increase of stress-related medication intake [15].

Workers already affected by high levels of stress are in an acute need of relief, and so waiting for the successful implementation of organizational changes can be challenging. In this situation, a stress management intervention (SMI) might be a first step to support those in need of help sooner [16]. There is evidence for the beneficial effects of SMIs in traditional face-to-face settings [17], which was complemented by a more recent and growing body of research for the web-based delivery [16,18,19]. Web-based interventions allow the workforce to benefit from low-threshold access and highly flexible participation in terms of time and location, and employers to profit from easy scalability and low required resources [20]. Furthermore, they might have the potential to alleviate the burden of workplace stressors by promoting self-efficacy and improving various health outcomes such as insomnia or depression [21-24] in both short and long term [25,26]. However, until today, evidence is missing on whether a web-based SMI could also effectively reduce perceived stress in employees who are exposed to adverse working conditions in terms of an imbalance between efforts and rewards. Moreover, no trial has yet examined mechanisms of change within this high-risk population and whether an increase in personal resources could enable employees to improve the unbalanced situation between efforts and rewards at work.

An effective implementation of a web-based SMI for employees who are exposed to adverse working conditions could be a person-centered intervention helping workers to initiate changes, a strategy known as problem-focused coping following the transactional model by Lazarus and Folkman [27]. A necessary personal resource for self-initiated changes employees make to redesign working conditions is self-efficacy, which is believed to trigger proactive behaviors undertaken at work [28]. Initially, Bandura [29] defined self-efficacy as confidence to meet difficult challenges or prospective problems by oneself. Individuals with high self-efficacy experience lower levels of work strain and engage more in problem-focused coping [30]. Another study confirmed that a problem-solving training for teachers could strengthen the ability to cope with problems and stressful situations as well as increase self-efficacy [31]. Within this organizational context, occupational self-efficacy can be described as personal belief in work-related abilities [32]. Studies on occupational self-efficacy have demonstrated positive associations with job performance, employee satisfaction, employability, and work commitment, and negative relationships with job insecurity [32,33]. A study on the same SMI that was examined in this randomized controlled trial (RCT) provided first evidence for effects on occupational self-efficacy [34], while the previously stated need for research on self-efficacy as a mechanism of change in an occupational SMI has not been addressed yet [35]. Moreover, there is no evidence on the effects of occupational self-efficacy on the perception of adverse working conditions yet despite the assumption that self-efficacy as a function of self-regulation conducive to health relies on successful exchange of efforts and rewards [36].

To the best of our knowledge, this is the first RCT to investigate the efficacy of a web-based occupational SMI in employees perceiving high stress levels and an effort-reward imbalance and to explore mediating effects of occupational self-efficacy, efforts, and rewards on stress reduction. This trial will examine the hypothesis that the SMI will effectively reduce perceived stress in the intervention group (IG) compared with a waitlist control (WLC) group. The second study aim is to investigate mediating effects of the personal resource of occupational self-efficacy and environmental factors, specifically efforts, and rewards at the workplace in the association between the intervention and perceived stress.

## Methods

### Study Design and Conditions

A primary RCT including 264 participants experiencing an effort-reward imbalance was conducted in compliance with the Declaration of Helsinki and Good Clinical Practice and following the CONSORT (Consolidated Standards of Reporting Trials) guidelines [37,38] (Multimedia Appendix 1). Based on meta-analytic evidence for web-based SMI revealing moderate effects (Hedges *g*=0.54) [19] and considering the impact of adverse working conditions, this study aimed to detect differences between groups with an effect size of Cohen *d*=0.35 based on a power (1–β) of 0.80 in a 2-tailed test with α=.05. Participants were randomly assigned to the IG or the WLC group at a ratio of 1:1 using an automated computer-based random integer generator (DatInf RandList; Datinf GmbH). Participants were allocated to the study groups by an independent researcher not otherwise involved in the study. Self-reported outcomes were assessed between May 2014 and May 2015 with a secured online-based self-report system (AES; 256-bit encrypted) at screening for eligibility (T0), baseline (T1), and 7 weeks (T2), and 6 months (T3) after randomization. After allocation, participants in the IG received immediate access to the intervention, whereas those in the WLC group obtained access after 6 months. Treatment as usual was not restricted and monitored. None of the obtained data presented here were published before.

### Participants and Recruitment

Participants were recruited from the general working population via the research project website and mass media (eg, articles in health insurance magazines). Inclusion criteria were the willingness to give informed consent; legal age (18 years); employment; 10-item Perceived Stress Scale (PSS-10) [39,40] score ≥22; effort-reward imbalance [41] score >0.715, which was found to indicate a highly hazardous imbalance between effort and rewards at the workplace [42]; no notable suicidal risk, as indicated by a score of >1 on item 9 (I feel I would be better off dead) of the Beck Depression Inventory [43]; and no previous or current diagnosis of dissociative symptoms or psychosis. Interested participants signed up on the open access website with their email address to receive a link to the eligibility screening questionnaire. Eligible applicants were required to provide informed consent and baseline data (T1).

### Intervention

Psychologists developed the intervention for employees based on Lazarus’ transactional model of stress focusing on problem solving and emotion regulation skills [27]. The intervention encouraged participants to reflect on meaningful issues that were not restricted to either work or personal life. The efficacy was demonstrated before in an indicated prevention sample and with different guidance formats, namely, adherence-focused guidance and self-help [22,44,45]. The SMI consisted of 7 core modules and an optional booster session 4 weeks after termination. Module completion required 45-60 minutes and participants were advised to complete at least one per week, adding up to an intervention period of 4-7 weeks. Participants could choose whether and how often they preferred to receive short automatic motivational SMS text messages to their mobile device (infrequent or intensive, ie, 1-3 SMS text messages daily). In addition, participants could inquire feedback-on-demand, which was provided by an e-coach within 48 hours only upon request on the internal messaging platform. E-coaches were skilled psychologists following feedback guidelines from the standardized manual for the intervention. Participants were assigned to an e-coach in a 1-to-1 ratio.

### Primary Outcome Measure

The primary outcome was perceived stress appraised with the German version of PSS-10 [39,40], which was also developed based on Lazarus’ transactional model of stress. The items assess to what extent participants experienced their lives as stressful within the past week on a 5-point Likert scale from 0 (never) to 4 (very often), resulting in sums from 0 to 40, with higher scores reflecting higher levels of stress. In this study, values for the internal reliability (Cronbach α) were .81 at T1, .89 at T2, and .92 at T3.

### Secondary Outcome Measures

Included measures for the secondary outcomes are listed in the following sections, with number of items, item range, and reliabilities assessed at T2.

#### Mediators

Among the secondary outcomes, 2 measures were assessed for the inclusion as mediators. First, the Effort Reward Imbalance Questionnaire Short Form [41] with the subscales *efforts* (3 items; α=.78) and *rewards* (7 items; α=.79; score range 1-4). And second, the short form of the Occupational Self-Efficacy Scale (OSS-SF [32]; 6 items; α=.89; score range 1-6).

#### Work-Related Health

The subscale *emotional exhaustion* of the Maslach Burnout Inventory (MBI-GS-D; 5 items; α=.87; score range 1-6) was used to evaluate work-related health [46]. The Utrecht Working Scale (UWES) [47] was used to examine work engagement (9 items; α=.93; score range 0-6). A single-item question was used to assess work ability (Work Ability Index) [48] and the Work Limitations Questionnaire [49] was administered to examine presenteeism.

#### Mental Health

The short version of the Centre for Epidemiological Studies’ Depression scale (CES-D) [50,51] was used to assess depression (15 items; α=.84; score range 0-3). The Connor-Davidson Resilience Scale [52] was used to examine resilience (10 items; α=.88; score range 0-4). The Assessment of Quality of Life (AQoL)-8D Multi-Attribute Utility Instrument [53] was used to examine health-related quality of life (35 items, different ranges from 1 to 5 and 1 to 6; α=.96) at T3.

#### Other Measures

To assess the level of satisfaction with the intervention, the Client Satisfaction Questionnaire adapted to web-based interventions was used (CSQ-I; 8 items; α=.92; score range 1-4) [54]. In addition, self-developed measures were used to assess demographics, current occupation, work sector, income, educational level, and previous use of health services.

### Statistical Analyses

Statistical analyses were performed according to the recommendations of the CONSORT statement [37]. Data were analyzed with SPSS Statistics version 25 (IBM Corp.) [55] based on the intention-to-treat principle. An additional per-protocol analysis was conducted for the primary outcome, including only participants who completed at least six modules. Analyses of covariance (ANCOVA) were calculated with outcome baseline scores as covariates and a 2-tailed significance level at P<.05 to detect between-group differences for the IG and the WLC group at T2 and T3. Simulation studies have already demonstrated the methodological robustness of ANCOVA against bias, higher precision, and statistical power for experimental studies [56,57]. To handle missing data, multiple imputations were conducted for the intention-to-treat and per-protocol analyses with 10 estimates for each value that were aggregated into an overall value [58].

### Response Analyses

The Reliable Change Index of Jacobson and Truax [59] was used to investigate improvements of the primary outcome on an individual level. The SD of 6.2 and the reliability of PSS-10 of the norm population [60] were used in the formula [1.96 × SD1 × sqrt(2) × sqrt(1–rel)] to calculate that a reduction in perceived stress could be defined as *reliably improved* if changes of more than ±5.16 points were detected from T1 to T2. Symptom-free status was achieved according to Jacobson and Truax [59] when participants scored more than 2 SDs below the baseline mean (T1) of the primary outcome in the IG (mean 23.76, SD 5.11). The number needed to treat and 95% CI were calculated to indicate the average number of participants who need to be treated to achieve an additional response compared with the control group [61].

### Mediation Analyses

Four mediation analyses were conducted using the PROCESS macro (version 4.0) for SPSS [62]. The models build up on each other to explore their individual and shared contribution in stress reduction. In all models, the independent variable (X) was the study condition, and the dependent variable (Y) was perceived stress (PSS-10 at T3). The proposed mediators were occupational self-efficacy at T2 (PROCESS model 4); occupational self-efficacy at T2 and efforts at T3 (PROCESS model 6); occupational self-efficacy at T2 and rewards at T3 (PROCESS model 6); and occupational self-efficacy, efforts, and rewards at T3 (PROCESS model 81). Baseline scores of the outcome and mediator were considered covariates. For indirect effects that were considered significant if P<.05 and 95% CIs did not cover 0, 10,000 bias-corrected bootstrap samples were applied [62]. An additional sensitivity analysis including only study completers was performed.

### Ethics Approval

The Ethical Committee of the Leuphana University of Lueneburg approved the study (reference Ebert201408_Stresstraining). The trial was registered in the German Clinical Trials Register (DRKS00005990).

## Results

### Participants and Baseline Characteristics

**Figure 1:**
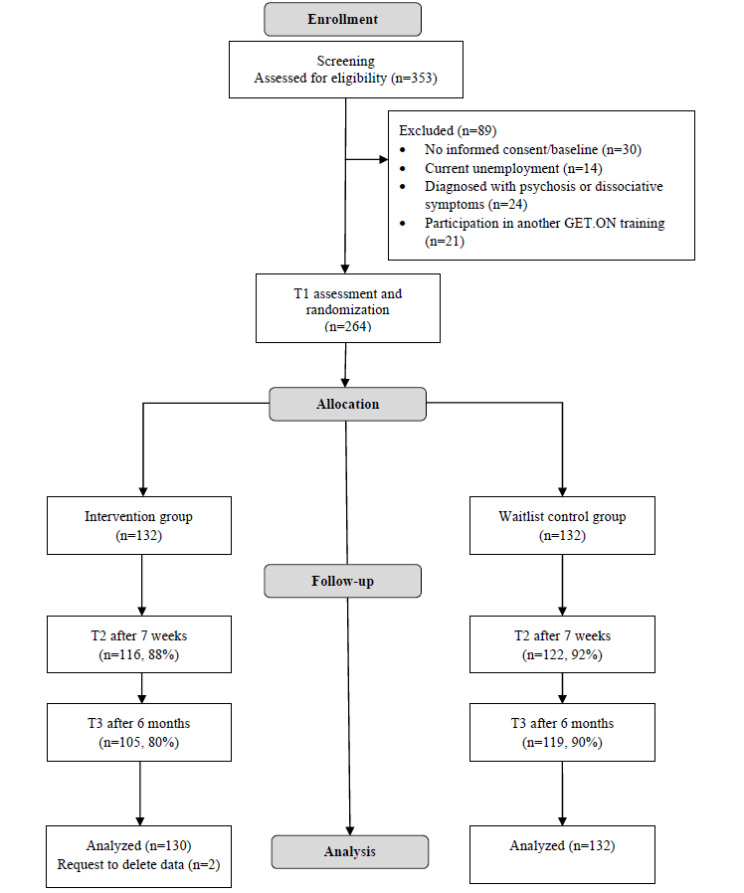
Participant flow.

The sample initially consisted of 264 participants of which 2 requested the deletion of assessed data after trial conduction. Consequently, the final sample included 262 participants (182/262, 69.4% female) aged 20-65 years (mean 42.2 years, SD 9.76 years), allocated to either the IG (n=130) or the WLC (n=132) group. Figure 1 depicts the study flow and Table 1 summarizes detailed baseline characteristics. A multivariate ANOVA indicated there was no meaningful difference in baseline outcomes between groups (*F*_19,232_=1.08, P=.37). Primary outcome data were missing for 9.9% (n=26) at T2 and 15.3% (n=40) at T3. The Little missing completely at random test failed significance, indicating that the null hypothesis proposing patterns of missing values being not dependent on observed and unobserved factors among the participants’ values need not be rejected.

**Table 1:** Baseline characteristics^a^.

Characteristics			All participants (N=262)		IG^b^ (n=130)		WLC^c^ group (n=132)
**Sociodemographic**							
	Age, mean (SD)	42.20 (9.76)		42.87 (9.54)		43.42 (10.02)	
	Men, n (%)	80 (30.5)		45 (34.60)		35 (26.5)	
	Women, n (%)	182 (69.5)		85 (65.40)		97 (73.5)	
	Diverse, n (%)	N/A^d^		N/A		N/A	
**Marital status, n (%)**							
	Single	78 (29.8)		41 (31.5)		37 (28.0)	
	Married	123 (46.9)		59 (45.4)		64 (48.5)	
	Cohabited	29 (11.1)		16 (12.3)		13 (9.8)	
	Divorced	31 (11.8)		14 (10.8)		17 (12.9)	
	Widowed	1 (0.4)		N/A		1 (0.8)	
**Educational level, n (%)**							
	Low	6 (2.3)		2 (1.5)		4 (3.0)	
	Middle	54 (20.6)		22 (16.9)		32 (24.2)	
	High	202 (77.1)		106 (81.5)		96 (72.7)	
**Employment**							
	Full-time, n (%)	205 (78.2)		105 (80.8)		100 (75.8)	
	Part-time, n (%)	53 (20.2)		23 (17.7)		30 (22.7)	
	Sick leave, n (%)	4 (1.5)		2 (1.5)		2 (1.5)	
	Managerial position, n (%)	100 (38.2)		50 (38.5)		50 (37.9)	
	Work experience in years, mean (SD)	18.40 (10.83)		17.79 (10.86)		19.01 (10.81)	
**Work sectors, n (%)**							
	Service	62 (23.7)		26 (20)		36 (27.3)	
	Economy	56 (21.4)		22 (16.9)		34 (25.8)	
	Health	33 (12.6)		22 (16.9)		11 (8.3)	
	Social	44 (16.8)		19 (14.6)		25 (18.9)	
	Information technologies	24 (9.2)		15 (11.5)		9 (6.8)	
	Other	36 (13.7)		19 (14.6)		17 (12.9)	
**Income, n (%)**							
	Low	73 (27.9)		29 (22.3)		44 (33.3)	
	Middle	45 (17.2)		26 (20)		19 (14.4)	
	High	123 (46.9)		64 (49.2)		59 (44.7)	
**Use of health services, n (%)**							
	Previous or current psychotherapy	119 (45.4)		55 (42.3)		64 (48.5)	
	Experience in health trainings	38 (14.5)		15 (11.5)		23 (17.4)	

^a^Values presented only for participants who provided the respective data.

^b^IG: intervention group.

^c^WLC: waitlist control.

^d^N/A: not applicable.

### Primary Outcome Measure

ANCOVAs to detect differences between the IG and the WLC group at T2 and T3 revealed significantly lower stress levels assessed with the PSS-10 for the IG at T2 (*F*_259,1_=46.14, P<.001, *d*=0.87, 95% CI 0.61-1.12, Δ5.00) and T3 (*F*_259,1_=24.82, P<.001, *d*=0.65, 95% CI 0.41-0.90, Δ4.19). The per-protocol analysis corroborated those results with significant between-group differences at T2 (*F*_173,2_=34.86, P<.001, *d*=1.04, 95% CI 0.69-1.40, Δ5.79) and T3 (*F*_173,2_=20.15, P<.001, *d*=0.56, 95% CI 0.22-0.90, Δ3.68). For all outcome measures at T2 and T3, Table 2 displays the means and SDs and Table 3 shows ANCOVA results.

**Table 2:** Means and SDs of outcome variables at baseline (T1), 7 weeks (T2), and 6 months (T3) after the intervention.

Outcome		T1			T2^a^			T3^a^	
		IG^b^	WLC^c^	IG		WLC	IG		WLC
**Primary outcome measure**									
	Perceived stress	23.76 (5.11)	24.81 (5.03)	18.33 (6.18)		23.33 (5.32)	17.53 (6.42)		21.72 (6.39)
	Perceived stress (per-protocol analysis)	23.89 (5.63)	24.79 (5.04)	17.5 (6.14)		23.29 (5.32)	18 (6.97)		21.68 (6.39)
**Secondary outcome measures**									
	Mental health and work related								
	Quality of life	0.58 (0.15)	0.55 (0.13)	N/A^d^		N/A	0.68 (0.17)		0.57 (0.17)
	Depression	17.05 (6.09)	18.05 (6.43)	13.68 (7.41)		15.76 (7.43)	11.56 (6.74)		14.8 (8.46)
	Resilience	20.12 (6.67)	20.29 (6.37)	22.5 (6.08)		19.38 (6.12)	N/A		N/A
	Emotional exhaustion	4.57 (0.78)	4.62 (0.73)	4.05 (0.89)		4.52 (0.81)	3.87 (0.94)		4.42 (0.95)
	Occupational self-efficacy	22.06 (6.14)	21.58 (6.24)	24.38 (5.41)		22.2 (6.08)	N/A		N/A
	Work engagement (vigor)	2.95 (1.21)	2.99 (1.18)	3.06 (1.17)		2.72 (1.15)	3.1 (1.18)		2.75 (1.21)
	Work engagement (dedication)	3.31 (1.3)	3.28 (1.37)	3.36 (1.17)		2.98 (1.36)	3.37 (1.27)		3.02 (1.3)
	Work engagement (absorption)	3.01 (1.39)	3.01 (1.51)	3.14 (1.28)		2.84 (1.38)	3.14 (1.27)		2.85 (1.38)
	Work ability index	5.92 (1.96)	5.86 (1.96)	6.55 (1.88)		5.83 (2.08)	N/A		N/A
	Presenteeism	5.01 (2.25)	5.27 (2.58)	4.5 (2.37)		4.94 (2.39)	N/A		N/A
	Effort-reward imbalance								
	Efforts	10.67 (1.42)	10.5 (1.52)	10.01 (1.76)		10.11 (1.76)	9.72 (1.76)		9.89 (1.83)
	Rewards	16.29 (3.74)	15.77 (3.86)	16.69 (3.8)		15.61 (3.96)	17.06 (3.62)		16.03 (3.9)
	Ratio	1.62 (0.49)	1.64 (0.49)	1.5 (0.55)		1.62 (0.56)	1.43 (0.47)		1.55 (0.54)

^a^Missing data handled by multiple imputation.

^b^IG: intervention group.

^c^WLC: waitlist control.

^d^N/A: not applicable.

**Table 3:** Between-group differences at 7 weeks (T2) and 6 months (T3) after the intervention.

Outcomes			T2^a^					T3^a^		
			*d* (95% CI)	ANCOVA^b^ (*F*_259,1_)			*d* (95% CI)			ANCOVA (*F*_259,1_)
**Primary outcome measure**										
	Perceived stress	0.87 (0.61 to 1.12)			46.14^c^	0.65 (0.41 to 0.90)			24.82^c^	
	Perceived stress (per-protocol analysis)^d^	1.04 (0.69 to 1.40)			34.86^c^	0.56 (0.22 to 0.90)			20.15^c^	
**Secondary outcome measures**										
	Mental health and work related									
	Quality of life^e^	N/A^f^			N/A	0.65 (0.37 to 0.93)			14.44^c^	
	Depression	0.28 (–0.04 to 0.52)			3.55	0.42 (0.18 to 0.67)			9.99^g^	
	Resilience	N/A			N/A	0.51 (0.26 to 0.76)			31.72^c^	
	Emotional exhaustion	0.56 (0.31 to 0.80)			25.36^c^	0.59 (0.34 to 0.83)			25.79^c^	
	Occupational self-efficacy	0.38 (0.13 to 0.62)			10.65^g^	N/A			N/A	
	Work engagement (vigor)	0.29 (0.05 to 0.53)			9.30^g^	0.29 (0.05 to 0.53)			7.61^g^	
	Work engagement (dedication)	0.30 (0.06 to 0.54)			8.71^g^	0.27 (0.03 to 0.52)			6.22^h^	
	Work engagement (absorption)	0.41 (0.66 to 0.17)			5.80^h^	0.22 (0.03 to 0.46)			3.97^h^	
	Presenteeism	0.18 (–0.06 to 0.43)			1.61	N/A			N/A	
	Work ability index	0.36 (0.12 to 0.60)			9.19^g^	N/A			N/A	
	Effort-reward imbalance									
	Efforts	0.05 (–0.19 to 0.30)			1.72	0.09 (–0.15 to 0.34)			1.87	
	Rewards	0.28 (0.04 to 0.52)			4.42^h^	0.27 (–0.03 to 0.52)			3.72	
	Ratio	0.22 (0.03 to 0.50)			4.21^h^	0.24 (0.01 to 0.48)			4.07^h^	

^a^Missing data handled by multiple imputation.

^b^ANCOVA: analysis of covariance

^c^Significance level used: P<.001.

^d^*F*_173,2_.

^e^*F*_200,1_.

^f^N/A: not applicable.

^g^Significance level used: P<.01.

^h^Significance level used: P<.05.

### Response Analyses

At T2, significantly more participants in the IG (65/130, 50%) showed a reliable improvement in perceived stress measured with the PSS-10 compared with the WLC group (33/132, 25%) and significantly fewer participants in the IG (4/130, 3.1%) experienced symptom deterioration compared with the WLC group (14/132, 10.6%; *χ*^2^_2_=19.94, P<.001). The number needed to treat to achieve reliable improvement was 4 (95% CI 2.8-7.3). The number of symptom-free participants at T2 was significantly higher in the IG (39/130, 30%) compared with the WLC group (7/132, 5.3%; *χ*^2^_1_=23.52, P<.001).

### Secondary Outcome Measures

The ANCOVAs showed significant between-group differences for most secondary outcome measures (Table 3). Positive impacts for participants in the IG compared with the WLC group were found at T2 and T3 for occupational self-efficacy (measured with the OSS-SF), burnout (assessed with the MBI-GS-D), work engagement (assessed with the UWES), and work ability (Work Ability Index). Effect sizes (*d*) ranged from 0.29 (95% CI 0.05-0.53; UWES scale vigor) to 0.56 (95% CI 0.31-0.80; MBI-GS-D) at T2 and from 0.22 (95% CI 0.03-0.46; UWES scale absorption) to 0.65 (95% CI 0.37-0.93; AQoL) at T3. Scores between groups did not significantly differ for depression (CES-D; *d*=0.28, 95% CI –0.04 to 0.52, P=.06) and work limitations (Work Limitations Questionnaire; *d*=0.18, 95% CI –0.06 to 0.43, P=.21) at T2. Regarding the effort-reward imbalance, participants in the IG showed significantly higher values for rewards at T2 (*d*=0.28, 95% CI 0.04-0.52, P=.04), whereas between-group scores did not significantly differ for efforts at T2 (*d*=0.05, 95% CI –0.19 to 0.30, P=.19) and T3 (*d*=0.09, 95% CI –0.15 to 0.34, P=.17), and for rewards at T3 (*d*=0.27, 95% CI –0.03 to 0.52, P=.06).

### Mediation Analyses

**Figure 2:**
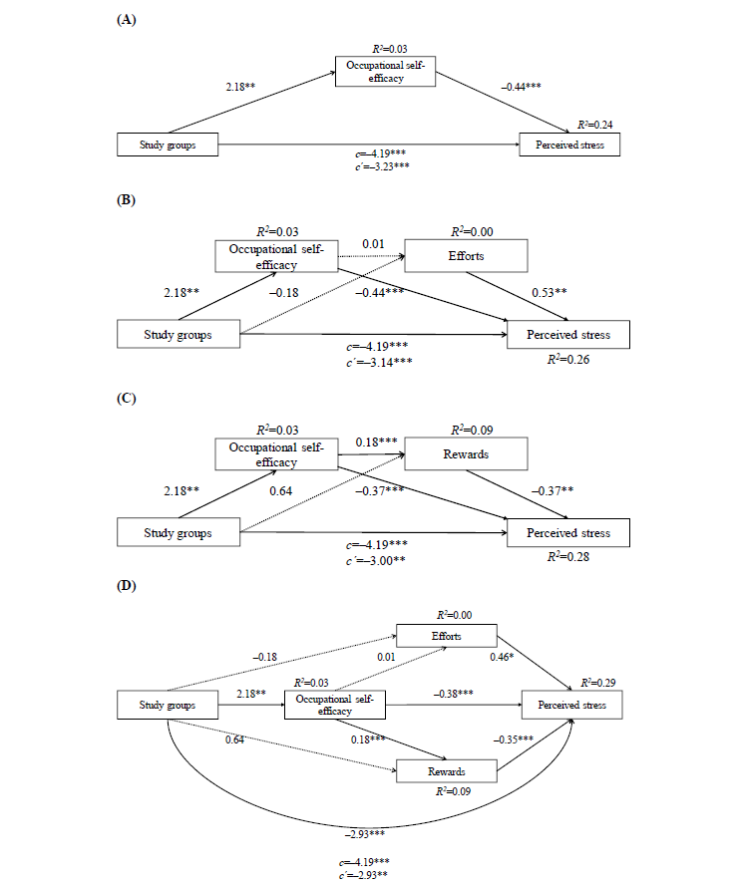
Mediation analyses with study condition as independent variable (X) and perceived stress (PSS-10) at T3 as dependent variable (Y) for all models. Proposed mediators: (A) Occupational self-efficacy at T2; (B) Occupational self-efficacy at T2 and efforts at T3; (C) Occupational self-efficacy at T2 and rewards at T3; and (D) Occupational self-efficacy at T2, and efforts and rewards at T3. Study conditions are coded 0=wait list control group, 1=intervention group. The figure includes unstandardized β coefficients and illustrates significant (solid line) and non-significant (dotted line) effects between variables, total (c) and direct (c′) effects. Significance levels used: ***P<.001, **P<.01, *P<.05.

Figure 2 depicts the 4 mediation analyses performed. Results of the first model (Figure 2A) showed that the unstandardized regression coefficient for the study groups (X) predicting stress (Y) was significant (*c*=–4.19, t_260_=–5.29, P<.001). Occupational self-efficacy (M) was found to be a significant mediator for this effect (*b*=–0.44, t_258_=–6.87, P<.001). Furthermore, the study group had a significant effect on occupational self-efficacy (*b*=2.18, t_260_=3.06, P<.002). The direct effect remained significant after incorporating the mediating variable into the model (*c′*=–3.23, t_260_=–4.36, P<.001). The indirect effect was significant (*b*=0.95, 95% CI –1.73 to –0.32, P<.001). This model accounted for 24% of the variance (*R*^2^) in stress reduction.

The second mediation model (Figure 2B) with occupational self-efficacy as M_1_ and efforts as M_2_ revealed significant total (*c*=–4.19, t_260_=–5.29, P<.001) and direct (*c′*=–3.14, t_260_=–4.36, P<.001) effects. Occupational self-efficacy (M_1_) significantly mediated the effect on stress (*b*=–0.44, t_258_=–6.59, P<.001), whereas it had no significant effect on efforts (M_2_; *b*=0.01, t_259_=0.27, P=.79). However, efforts (M_2_) were significantly associated with stress (*b*=0.53, t_258_=2.64, P=.008). The study group had no significant effect on efforts (M_2_; *b*=–0.18, t_258_=–0.79, P=.43). Therefore, a significant indirect mediating effect was only found for the association of occupational self-efficacy with the study group (*b*=–0.14, 95% CI –0.25 to –0.05, P<.001). Together, 26% of the variance (*R*^2^) in perceived stress was explained.

After incorporating occupational self-efficacy as M_1_ and rewards as M_2_, the mediation model (Figure 2C) resulted in significant total (*c*=–4.19, t_260_=–5.29, P<.001) and direct (*c*′=–3.00, t_260_=–4.12, P<.001) effects. Occupational self-efficacy (M_1_) significantly mediated the effect on stress (*b*=–0.37, t_258_=–5.76, P<.001) and rewards (M_2_; *b*=0.18, t_259_=4.52, P<.001). Rewards (M_2_) could significantly predict stress (*b*=–0.37, t_258_=–3.79, P<.001). Comparable to the preceding mediation model, a significant indirect mediation effect for the association between the intervention and stress as an outcome could be found for occupational self-efficacy (*b*=–0.12, 95% CI –0.22 to –0.04, P<.001). Furthermore, the indirect path taking occupational self-efficacy (M_1_) and rewards (M_2_) between the study group and perceived stress into account was significant (*b*=–0.02, 95% CI –0.05 to –0.01, P<.001). Participation in the intervention did not significantly predict rewards (M_2_; P=.16). In total, all variables accounted for *R*^2^=0.28.

The fourth mediation model (Figure 2D) that incorporated all mediators (M_1_: occupational self-efficacy, M_2_: efforts, and M_3_: rewards) again resulted in significant total (*c*=–4.19, t_260_=–5.29, P<.001) and direct (*c*′=–2.93, t_260_=–4.06, P<.001) effects. Occupational self-efficacy (M_1_) significantly predicted perceived stress (*b*=–0.38, t_257_=–5.91, P<.001) and rewards (M_3_) (*b*=0.18, t_259_=4.52, P<.001), yet not efforts (M_2_; *b*=0.01, t_259_=0.27, P=.79). The effect on stress was also significantly predicted by both efforts (M_2_; *b*=0.46, t_257_=2.32, P=.02) and rewards (M_3_; *b*=–0.35, t_257_=–3.56, P<.001). The study group did not significantly predict neither efforts (M_2_) nor rewards (M_3_) directly. Altogether, significant indirect paths between the study group and perceived stress were found for occupational self-efficacy (M_1_; *b*=–0.12, 95% CI –0.22 to –0.04, P<.001) as well as for occupational self-efficacy (M_1_) and rewards (M_3_; *b*=–0.02, 95% CI –0.05 to –0.01, P<.001). This final model including all proposed mediators together explained 29% of the variance (*R*^2^) in stress reduction. For all models, sensitivity analyses performed including only study completers corroborated the results.

## Discussion

### Principal Findings

Results of this study confirm that the SMI could effectively reduce stress in employees perceiving elevated stress levels and even when they were exposed to a high load of efforts that is not adequately balanced by rewards. Secondary analyses demonstrated the beneficial effects for mental health and work-related outcomes as well as for rewards. Step-by-step mediation analyses revealed that the participation in the intervention significantly predicted occupational self-efficacy, which describes the confidence of an individual to handle any challenges at work and which was a mediator in the effect on stress and rewards that again predicted stress. All 3 investigated mediators (ie, occupational self-efficacy, efforts, and rewards) were significantly associated with perceived stress. However, neither participation in the SMI nor the increase in occupational self-efficacy enabled employees to achieve favorable effects on the level of efforts, while efforts still enfolded an adverse effect on perceived stress.

The results revealed practically meaningful effect sizes for stress reduction. A similar effect was found in another trial on the same SMI with adherence-focused guidance [45] and our study extend those results by the inclusion of a high-risk population that experiences adverse working conditions. Compared with a study on the same SMI with more intensive guidance [22], the effect sizes were not as large at follow-up. This raises the question as to whether more personal support from a mental health expert, which is expected to be conducive to the efficacy of an SMI [19,22], might aid participants that experience greater difficulties in their stress management due to adverse working conditions. Considering the efficacy of occupational web-based interventions in general, results from this study are in line with demonstrated average effect sizes in a recent meta-analysis [19] and revealed significant improvements in a variety of outcomes on mental health and work-related levels. For example, participants in the IG showed lower levels of emotional exhaustion, more resilience, and higher work engagement, as well as vigor, dedication, and absorption at work. No significant between-group effects were found for presenteeism, while mixed results were obtained for depression. The detected effect sizes for engagement and presenteeism compare with a recent meta-analysis for occupational web-based interventions [63]. Moreover, the participation significantly increased occupational self-efficacy that was shown to be a relevant mediator in the efficacy of the SMI on stress reduction. These results support findings of another RCT on the same SMI showing significant effects on occupational self-efficacy [34] and positive associations between stress levels and self-efficacy [30]. The obtained results for the effort-reward imbalance tie well with mixed effects found in studies on the same SMI for the effort-reward imbalance ratio [34] and for efforts and rewards evaluated as separate outcomes [45], demonstrating that web-based SMIs enfold substantially larger effects on individuals’ health compared with perceived working conditions and organizational characteristics [17].

To examine whether and how an increase in personal resources could support participants in achieving successful stress reduction despite facing adverse working conditions, mediating effects were investigated not only for occupational self-efficacy, but also for efforts and rewards of the workplace. The 4 mediation analyses conducted progressively accounted for the variance in perceived stress. The first model (Figure 2A) confirmed that the participation in the intervention successfully increased occupational self-efficacy, which in turn had a significant effect on stress reduction. This is in line with evidence showing that higher levels of self-efficacy are associated with lower levels of work stress and the assumption that problem-solving skills increase the confidence of an individual to be able to proactively reduce stressors and increase rewarding situations [30]. The second mediation model (Figure 2B) showed that the intervention’s positive effect on occupational self-efficacy did not affect efforts that were negatively associated with stress. This is in line with another SMI study on teachers which showed that participants could influence rewards, yet not efforts [64]. One potential reason for the lack of association could be the design of the intervention that did not predefine the topics participants should reflect on in the problem-solving exercises and if the focus was on job-related or personal stressors. Furthermore, this portrays one of the core premises of the effort-reward imbalance model [6], that is, an increased degree of efforts necessary to spend at work is associated with high strain. The third mediation analysis (Figure 2C) revealed a significant relationship between participation in the SMI, occupational self-efficacy, and rewards. This is in line with evidence showing that occupational self-efficacy is substantially associated with affective commitment that might motivate employees to increase their job resources within their company [65]. Comparable to the precedent mediation model, rewards were significantly associated with stress, which is in line with the effort-reward imbalance model [6]. The final mediation analysis (Figure 2D) incorporated the 3 models. Occupational self-efficacy was significantly increased and a mediator in the relationship between the study group and outcome. Although both efforts and rewards predicted levels of stress, the intervention only had an impact on rewards, but not on efforts, with occupational self-efficacy seemingly playing a mediating role in this association. However, both efforts and rewards had significant effects on stress.

### Limitations

Several limitations should be considered. Despite the positive effects of the individual-focused intervention on employees’ mental health, the persisting adverse effects of efforts indicate that this approach might be incomplete. Therefore, it should be investigated whether a combination of individual- and organizational-focused digital interventions will contribute to more comprehensive effects on employees’ mental health [66]. Positive effects of occupational self-efficacy in individual-focused interventions might help employees to engage more confidently in organizational-focused interventions. Furthermore, the generalizability of the results might be limited. In contrast to recruitment on a company level, the applied open recruitment strategy addressed participants directly, which was shown to be associated with effects on personal health outcomes for occupational SMIs [19]. In this study, participants in the IG received adherence-focused guidance that was established and shown to be effective in previous studies [45,67]. Given the notion that guidance is supposed to be conducive to the efficacy of SMIs [19,68] and its low intensity in the adherence-focused format, further research could investigate whether a higher intensity in guidance might facilitate the efficacy of the SMI for participants that experience greater difficulties for successful changes due to adverse workplace conditions. Concerning the mediators, a methodological limitation might be the selection of measures in this study because participants might have been encouraged to make changes to aspects of their work that were not captured in this trial (eg, conflict between work and private life) [69]. Despite this, this trial provides valuable first insights into if and how a web-based SMI can be effective within a high-risk population despite their exposure to adverse working conditions.

### Conclusion and Practical Implications

To conclude, this trial aimed to expand research on the efficacy of web-based SMIs and to add valuable insights into the scarce evidence for high-risk populations. To the best of our knowledge, this is the first trial demonstrating positive effects of a web-based SMI on stress reduction in employees despite their adverse working conditions. In-depth analyses examining mechanisms of change suggest that the SMI increased occupational self-efficacy that mediated the intervention’s effect on stress. Furthermore, both efforts and rewards predicted levels of stress, yet the intervention only had an impact on rewards, with occupational self-efficacy seemingly playing a mediating role in this association. It seems vital to note that this web-based intervention could improve health at work within a short period and without any direct changes to working conditions. Further medium- and long-term improvements would be possible if complex organizational interventions were introduced to reduce stressors in the workplace. For practice, these results have several implications. First, the implementation of the web-based SMI can be recommended due to its beneficial health effects even if employees experience adverse working conditions. Second, occupational self-efficacy should be considered as an important concept in the design of an SMI. Third, the limited effects of the SMI on the perception of working conditions underline that organizational top-down changes are still indispensable. Future studies could further investigate which factors contribute to the efficacy of a person-centered intervention on working conditions and examine, for example, the role of guidance.

## Appendix

Multimedia Appendix 1 CONSORT-eHEALTH checklist (V 1.6.1).

## Abbreviations

ANCOVA: analysis of covariance
AQoL: Assessment of Quality of Life 8D Multi-Attribute Utility Instrument
CES-D: Centre for Epidemiological Studies’ Depression Scale
CONSORT: Consolidated Standards of Reporting Trials
IG: intervention group
MBI-GS-D: Maslach Burnout Inventory
N/A: not applicable
OSS-SF: short form of the Occupational Self-Efficacy Scale
PSS-10: 10-item Perceived Stress Scale
RCT: randomized controlled trial
SMI: stress management intervention
UWES: Utrecht Work Engagement Scale
WLC: waitlist control
WLQ: Work Limitations Questionnaire
